# Efficacy of Manual Wheelchair Skills Training for Improving Skills and Confidence in People With Hereditary Degenerative Disorders: Protocol for a Sequential Multimethods Study

**DOI:** 10.2196/66974

**Published:** 2025-07-31

**Authors:** Ernest Niyomwungere, François Routhier, Cynthia Gagnon, R Lee Kirby, Xavier Rodrigue, Isabelle Lessard, Josiane Lettre, Krista L Best

**Affiliations:** 1 School of Rehabilitation Sciences Université Laval Quebec City, QC Canada; 2 Centre for Interdisciplinary Research in Rehabilitation and Social Integration Centre Intégré Universitaire de Santé et de Services Sociaux de la Capitale-Nationale Quebec City, QC Canada; 3 École de Réadaptation Université de Sherbrooke Sherbrooke, QC Canada; 4 Groupe de Recherche Interdisciplinaire sur les Maladies Neuromusculaires Centre Intégré Universitaire de Santé et de Services Sociaux du Saguenay–Lac-Saint-Jean Jonquière, QC Canada; 5 Division of Physical Medicine & Rehabilitation Department of Medicine Dalhousie University Halifax, NS Canada; 6 Physiatry Department Centre Intégré Universitaire de Santé et de Services Sociaux de la Capitale-Nationale Quebec City, QC Canada; 7 Centre d’Étude des Conditions de Vie et des Besoins de la Population Cégep de Jonquière Jonquière, QC Canada

**Keywords:** ARSACS, myotonic dystrophy type 1, manual wheelchair, wheelchair skills training, mobility, participation, randomized controlled trial

## Abstract

**Background:**

Mobility impairment and participation restrictions are commonly experienced by individuals with autosomal recessive spastic ataxia of Charlevoix-Saguenay (ARSACS) and myotonic dystrophy type 1 (MD1), 2 disorders that are highly prevalent in the province of Quebec, Canada. People with ARSACS and MD1 experience a progressive decline in mobility, which commonly results in the provision of manual or power wheelchairs. While wheelchairs can facilitate mobility and social participation, their provision alone does not guarantee safe and effective use. Wheelchair skills training has been shown to be effective for improving manual skills and confidence among adult users with various diagnoses, which may enhance self-directed mobility and participation and reduce the risk of chronic and acute injuries. However, manual wheelchair skills training for people with ARSACS and MD1 remains understudied.

**Objective:**

The primary aim of this study is to evaluate the efficacy of manual wheelchair skills training for safely improving wheelchair performance in people with ARSACS and MD1. The secondary outcomes include exploring the influence of manual wheelchair skills training on skill capacity, use self-efficacy, mobility, and the retention of outcomes 3 months later. We will also qualitatively explore the manual wheelchair training experiences of people with ARSACS and MD1.

**Methods:**

This study will use a sequential multimethods design, combining a waitlist randomized controlled trial and qualitative interviews. The participants will include adults who have a diagnosis of ARSACS or MD1 who use a manual wheelchair for mobility. Participants will be randomly assigned to the intervention or control group using a 1:1 allocation ratio. The intervention group will receive 5 manual wheelchair skills training sessions (1-2 sessions/week), while the control group will receive no training. Data will be collected at baseline (T1), after the 4-week intervention (or waiting period for the control group; T2), and 3 months after T2 to assess retention (T3). The primary outcome will be manual wheelchair skills performance. Secondary outcomes will include manual wheelchair skills capacity, self-efficacy, and mobility. Semistructured individual interviews will be conducted to explore participants’ expectations regarding manual wheelchair use, past manual wheelchair experiences, and perceptions of manual wheelchair skills training. Quantitative data will be analyzed using analysis of covariance (ANCOVA), controlling for baseline scores, and qualitative data will be analyzed using reflexive thematic analysis.

**Results:**

This study received ethical approval (2025-3100) in July 2024. Recruitment started in January 2025. A graduate student, a research assistant, and a research coordinator have been recruited and trained.

**Conclusions:**

The results of this randomized waitlist-controlled trial will confirm whether manual wheelchair skills training can improve self-directed mobility and related outcomes for people with ARSACS and MD1. The findings may help guide clinical practice toward manual wheelchair skills training for understanding potential influences on manual wheelchair mobility in people living with neuromuscular disorders.

**Trial Registration:**

ClinicalTrials.gov NCT06596850; https://clinicaltrials.gov/study/NCT06596850

**International Registered Report Identifier (IRRID):**

PRR1-10.2196/66974

## Introduction

Mobility impairment and participation restrictions are commonly experienced by people with neuromuscular and hereditary degenerative disorders [1]. Two highly prevalent disorders in the province of Quebec include autosomal recessive spastic ataxia of Charlevoix-Saguenay (ARSACS) [2,3] and myotonic dystrophy type 1 (MD1) [4,5]. Declining mobility is a hallmark among people with ARSACS and MD1 that can negatively influence physical and psychosocial health outcomes. Approximately 74% of individuals with MD1 have mobility impairments that restrict participation in activities of daily living, leisure activities, and vocation [6-8].

Wheelchairs (manual or power) are prescribed to facilitate mobility for people with ARSACS and MD1 when walking becomes difficult. In the reported literature, approximately 45% of individuals with ARSACS use wheelchairs [9-11], with the average age of wheelchair provision at 38.9 years [10]. Given the varying rate of progression of ARSACS, wheelchairs may be suggested during adolescence or older age (eg, 17 to 59 years of age) [12], but most people (if not all) will eventually need one for mobility. A patient registry of MD1 in the United Kingdom reported that 56% of patients used a wheelchair, with the average age at which wheelchair use began being 34.6 years, ranging from infancy to old age (1 to 72.4 years). Among the wheelchair users in this registry, 22.8% started using it before the age of 10 years and 56.6% began between 30 and 59 years of age [13].

Regardless of diagnosis or type of wheelchair (ie, manual or power), wheelchair provision alone does not guarantee safe and effective use. If not used adequately, wheelchairs could be associated with mobility dependence, restricted participation, reduced social connectedness [14], and reduced quality of life [15]. Moreover, unsafe wheelchair use could lead to accidents that vary in severity from cuts and bruises to life-threatening head injuries [16]. Furthermore, injuries due to wheelchair accidents may increase caregiver burden and strain on the health care system [17].

Learning how to use the wheelchair and developing appropriate wheelchair skills are critical to independent mobility and participation [18]. However, wheelchair mobility for people with ARSACS and MD1 remains understudied. Our team recently demonstrated that adults with ARSACS had lower manual and power wheelchair skills but fewer training goals than adults with other diagnoses (eg, spinal cord injury) [19]. This disparity may be attributed to limited training opportunities and low expectations of wheelchair use for individuals with ARSACS [19]. It may be that the progressive nature of ARSACS and MD1 (and other neuromuscular disorders) and the clinical focus on maintaining functional walking have historically taken clinical precedence over wheelchair mobility outcomes.

The World Health Organization suggests training in wheelchair use and follow-up as important steps in wheelchair service provision [20]. Four meta-analyses demonstrate the effectiveness of wheelchair skills training for improving wheelchair skills, use confidence, and participation [20-23]. However, a survey of 68 Canadian rehabilitation centers demonstrated that >50% of therapists spent 2 hours or less on wheelchair skills training and 18% provided no training at all [24]. Moreover, individuals with ARSACS and MD1 do not typically spend periods of time in rehabilitation centers where most wheelchair skills training occurs. The inherited and progressive nature of ARSACS and MD1 means that people receive wheelchairs when they can no longer walk but do not commonly receive additional assessments, training, or follow-ups related to wheelchair use. Given the evidence on the effect of manual (manual wheelchair) skills training in other progressive conditions (eg, multiple sclerosis, Parkinsonism) [22,23], the association between manual wheelchair mobility and the preservation of upper limb function and strength in ARSACS [19], it is likely that evidence-based training could improve manual wheelchair skills, confidence, and social participation for people with ARSACS, MD1, and other neuromuscular disorders. In a pilot study, 3 individuals with ARSACS improved their manual wheelchair skills (between 18% and 30%) and confidence (between 5% and 15%) after 3 hours or less of manual wheelchair skills training [25].

The overall aim of this study is to evaluate the efficacy of an evidence-based manual wheelchair skills training program for improving safe manual wheelchair mobility in people with ARSACS and MD1. The primary objective is to test the hypothesis that participants who receive manual wheelchair skills training will improve their manual wheelchair performance by at least 20% compared to those in a control group who receive no training. The secondary objectives are to explore the influence of manual wheelchair skills training on wheelchair skills capacity, use self-efficacy, and mobility. Finally, the participants’ expectations regarding manual wheelchair use and the experiences and perceived impact of manual wheelchair skills training on mobility and quality of life will be explored using a qualitative inquiry.

## Methods

### Study Design

A sequential multimethods randomized controlled trial (RCT) and qualitative interviews will be used, with the RCT reported according to the CONSORT (Consolidated Standards of Reporting Trials) guidelines [26]. Participants will be randomly assigned to the intervention or control group using a 1:1 allocation ratio. A randomization process with undisclosed block size will be created by a statistician who is not otherwise involved in the project. Given the potential benefits of the intervention already reported in our previous studies [22,23], control group participants will also receive wheelchair skills training following a 4-week control period and postintervention data collection (ie, they will be on a “waitlist” for the wheelchair skills training program and receive no intervention during the study period).

### Ethical Considerations

The protocol for this study was approved by the Research Ethics Boards in rehabilitation and social integration of the Centre Intégré Universitaire de Santé et de Services Sociaux de la Capitale-Nationale (CIUSSS-CN; 2025-3100). The study protocol was registered on ClinicalTrials.gov (NCT06596850). Informed consent will be obtained from all participants by a research coordinator (RC). Participants will be made aware that they can withdraw from the study at any moment without facing any consequences. All collected information will remain strictly confidential within the limits established by law. To protect privacy and confidentiality, participants will be identified by a unique code, and their data will be anonymized and securely stored on a password-protected server. Participants will receive financial compensation for data collection activities to acknowledge their time and reduce attrition (CAD $50, equivalent to US $36, after each assessment session).

### Participants and Recruitment

Convenience sampling will be used to recruit 20 community-dwelling people with ARSACS or MD1 who use manual wheelchairs. Active recruitment strategies will include recruiting through outpatient services at the local rehabilitation center in Quebec City (CIUSSS-CN). The chief of physiatry (author XR) who has clinical expertise in ARSACS and MD1 will discuss the study with all individuals on his case load who have a diagnosis of ARSACS or MD1 and use manual wheelchairs. In addition, participants from a longitudinal study on ARSACS (completed in 2022) will be contacted and informed of the study. Passive recruitment strategies will include disseminating promotional videos on the social media and websites of Muscular Dystrophy Canada, other collaborators, and community organizations. Participants will be 18 years of age or older; have a diagnosis of ARSACS or MD1; use a manual wheelchair for mobility (≥ 3 times/week for ≥ 8 hours/week); and be able to propel their manual wheelchair at least 10 meters. Individuals will be excluded if they anticipate a health condition or procedure that contraindicates training (eg, surgery) or if they are concurrently receiving or planning to receive other manual wheelchair training during the study.

### Sample Size

The sample size was powered to detect a statistically significant difference of 20% between groups using mean change scores from baseline to postintervention in wheelchair skills [27]. A large effect size (Cohen *d* =1.2) for the wheelchair skills training and a correlation between measures (*R*^2^) of 0.6 was used. With a significance level of α=.05 and 90% power, a sample size of 18 was required for a repeated measures analysis of covariance (ANCOVA) (*F* tests) with between-group factors, controlling for baseline score (including function and wheelchair experience), as calculated using G*Power [28]. Adjusting for a 10% loss to attrition, a total sample of 20 is required (10 per group).

### Study Procedures

**Figure 1:**
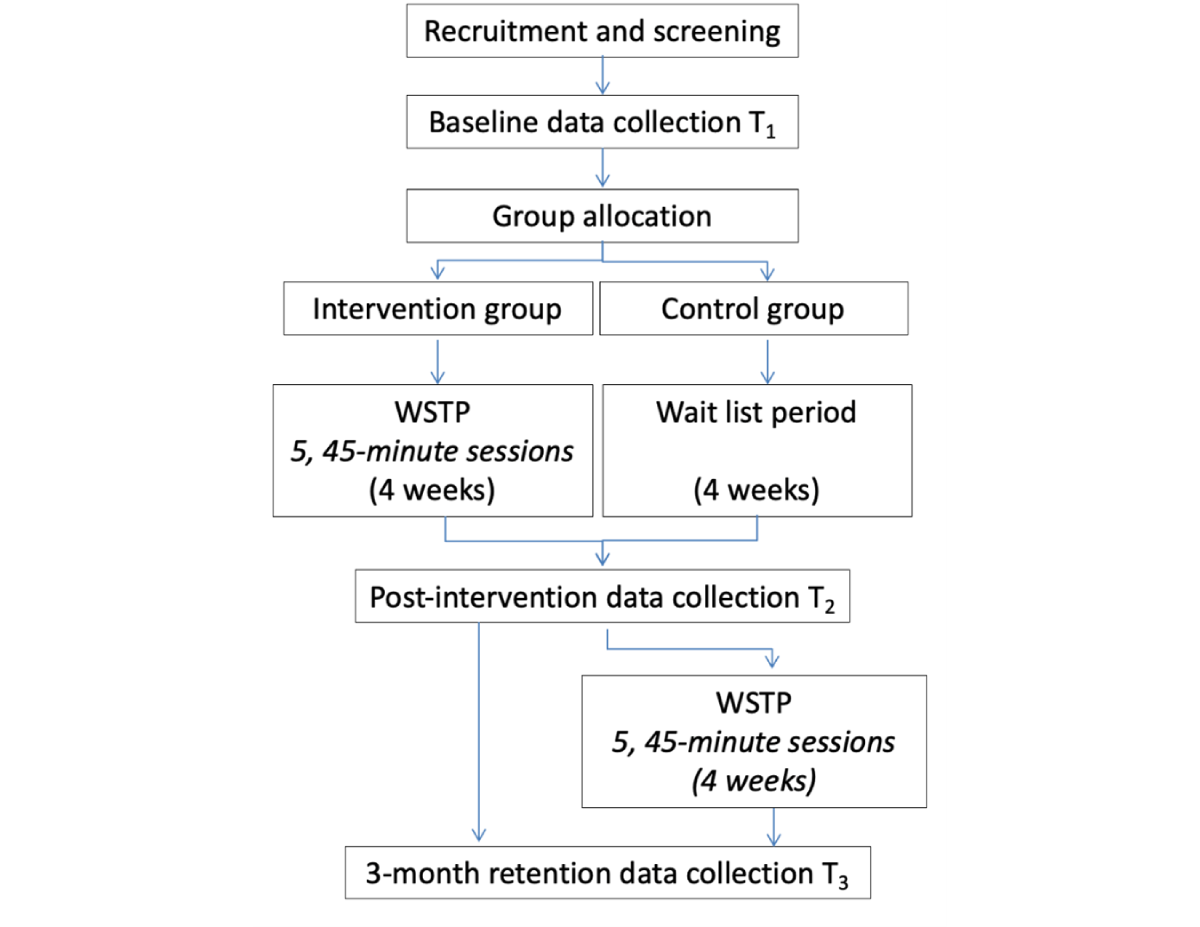
Trial design describing coordination, testing, and experimental procedures. WSTP: Wheelchair Skills Training Program.

Following enrollment, participants will complete baseline measures (T1) and then be assigned to either the experimental or the control group. An RC will then contact the statistician via telephone or email to obtain the group assignment within 48 hours. The participant will be informed of the group assignments, and the trainer will be given the participants’ contact information to schedule the training sessions and assign the control group. The training will be scheduled after completing the blinded postintervention data collection (T2). In an effort to minimize bias, a research assistant (RA) will be blinded to group allocation and participants will be instructed not to discuss their program with the RA. The RC will also arrange for the blinded follow-up (retention) data collection for all participants 3 months after postintervention data collection (T3). Qualitative interviews will be conducted by the RC by phone or videoconferencing within 7 days of completing the intervention. Given the self-report nature of the primary outcome, the interdisciplinary experience of 3 study investigators with RCTs who are not located at the data collection site, and the involvement of a citizen partner and Muscular Dystrophy Canada in data collection, analysis, and interpretation, a data monitoring committee was not established. Participants in the intervention and control groups will continue to receive usual care (if any) during the study. The trial design describing coordination, testing, and experimental procedures is depicted in Figure 1.

### Intervention Details

According to the evidence-based Wheelchair Skills Training Program (WSTP) [29], participants will receive 5 individual 45-minute weekly wheelchair skills training sessions. As previously described by Best et al [26], the training sessions will take place in the community, in and around the participants’ homes and neighborhoods (eg, libraries, shopping centers, museums, and parks), and will be customized according to the participants’ goals. The WSTP provides suggestions for individualizing the approach based on the learner’s impairments (ie, weakness, spasticity, contractures, cognitive impairments, and movement disorders like ataxia, tremor, and chorea). Therefore, specific training considerations can be made regardless of diagnosis. The WSTP sessions will begin with a 5-minute review of goals/progress, followed by a 10-minute warm-up (wheeling activities, random practice of previously learned skills). This will be followed by 20 minutes of attempting new skills, with training on each skill carried over to the next session until the skills are learned or the trainer and participant mutually agree to stop training. The trainer will periodically ask the participant to practice newly learned skills to incorporate variability of practice. Finally, a 10-minute cooldown will take place, during which the participant will practice skills in a self-controlled environment.

The trainer (author EN), a graduate student with training in physiotherapy will receive two 4-hour sessions by experts in manual wheelchair skills training (authors KLB, FR, and RLK), which has been shown to improve knowledge and confidence for training wheelchair skills [30]. The trainer will also complete self-guided learning modules, semistructured practice with trained trainers, and the Wheelchair Skills Program Certification Exam [31]. The fidelity of the WSTP administration will be evaluated by a member of the study team not involved in testing or training who will attend 1 training session per participant (randomly throughout the training period). The trainer will also complete a detailed training log, a checklist of standardized steps to be followed during training, and document adherence to the intervention and any adverse events. Formative assessments with the trainer will ensure fidelity.

### Control Group

Participants in the control group will receive the usual care (if any) during a 4-week period. During this time, participants will be considered on a waitlist to receive the WSTP after the completion of the project and may continue or begin any activities provided by therapists or in the community. After completing the T2 evaluations, participants in the control group will complete a target of five 45-minute weekly sessions of the WSTP, delivered by trained personnel as described for the intervention group. While usual care may include some form of manual wheelchair skills training, the evidence suggests that the amount of training received during usual care, according to the Wheelchair Skills Program, is limited [24].

### Data Collection

The RA, an occupational therapist with 12 years of experience in clinical research, will collect quantitative data and enter them into a secure database. Sociodemographic information will include age, sex, gender, diagnosis, length of time using a wheelchair, previous experience with using a manual wheelchair (where and for which activities), previous wheelchair training, and assistance required for using a manual wheelchair. Assistance will be assessed using the 5-point General Scale for the Extent of Caregiver Assistance scale (5=no assistance/always autonomous, 4=standby assistance only, 3=verbal assistance only, 2=one-person physical assistance, 1=two-person physical assistance, and -0=equipment needed [31]. Additional information will include the propulsion method and wheelchair specifications (frame type, dimensions, tire, and caster size).

### Primary Outcome

Manual wheelchair skills performance (ie, what the person does in their daily life) will be assessed using the Wheelchair Skills Test Questionnaire for Manual Wheelchair Users (WST-Q-M version 5.4.2) [32,33]. The WST-Q-M comprises 30 discrete skills required to move around in a manual wheelchair indoors and in the community. Participants will rate their performance for each of the 30 manual wheelchair skills on a 4-point scale. The root performance question asks, “Could you do this (manual wheelchair skill) in your own setting?” Response options include “yes, very well (3),” “yes, but not well (2),” “yes, with help (1),” “no (0),” and “no part” (NP) with the manual wheelchair. The total percentage WST score is calculated as: total percentage WST performance=sum of individual skill scores/([# of possible skills – # of NP scores – # of testing error (TE) scores] 3) × 100%. Participants will be asked whether the skill represents a training goal (yes, no) to assist with defining the training program. The WST-Q-M can be completed in approximately 15 minutes, has strong psychometric properties in English and French [34,35], and has been used extensively in clinical trials [16]. The WST-Q-M has been validated by the study team for ARSACS [36]. A minimally clinically important difference (MCID) of 20% relative improvement in WST scores (T1 to T2) has been suggested for adults [22]. However, a recent invited commentary by Best and Kirby [37] suggests that the potential clinical significance of much smaller improvements should be considered.

### Secondary Outcomes

Manual wheelchair skills capacity (ie, what a person can do in a standardized environment) will be assessed using the Wheelchair Skills Test (WST) v5.4 for manual wheelchair users [38,39]. Participants will be asked to execute 30 manual wheelchair skills in a standardized environment. They will be scored on a 4-point scale as “advanced pass (3),” “pass (2),” “partial pass (1),” “fail (0),” “NP,” or “TE.” The total percentage WST score is calculated as: total percentage WST capacity score = sum of individual skill scores/([# of possible skills – # of NP scores – # of TE scores] × 3) × 100%. The WST has been validated in adult populations. An MCID of 20% relative improvement in WST scores (T1 to T2) has been suggested for adults [22].

Manual wheelchair use self-efficacy (ie, belief in one’s ability to accomplish specific tasks while using a manual wheelchair) will be assessed using the Wheelchair Use Confidence Scale for Manual Wheelchair Users Short Form (WheelCon-M) [40]. WheelCon-M comprises 21 statements related to confidence using a manual wheelchair in activities and environments, each rated on a scale from 0 (“not at all confident”) to 10 (“completely confident”), producing a total mean score of 0 to 10 [40,41]. Responses will indicate the current level of perceived confidence in 6 areas (ie, navigate the physical environment in a manual wheelchair, perform activities in a manual wheelchair, problem solve, advocate for needs, and manage social situations and emotions). The WheelCon-M has been validated for ARSACS [36].

Manual wheelchair mobility will be measured using actigraphy, collected with a small, noninvasive, and lightweight accelerometer (Actigraph GT3X; Ametris). The Actigraph GT3X contains a multidirectional accelerometer that integrates information about direction and speed to produce an electrical current with variable magnitude and duration, and the electrical current data are stored as activity counts [42]. The time between sampling units (epochs) will be set at 15 seconds [42].

The participants will receive 2 actigraphs after completing the assessments (T1, T2, and T3); one will be worn on their nondominant arm between the elbow and shoulder, and one will be placed on the rear wheel of their manual wheelchair inside a custom-made waterproof packet that will be installed using tie-wraps. Participants will be asked to wear the actigraphs at all times during a 7-day period, except during sleep, bathing/showering, or swimming. They will also be asked to log the exact time the actigraph was put on and taken off each day [43]. The tester will obtain the actigraphs and logs from the participants at the end of the 7-day period (either in person or by postage-paid envelopes that will be provided to the participants). Actigraphy has been validated for evaluating manual wheelchair mobility in adults [44].

### Qualitative Interviews

The participants will complete semistructured individual interviews lasting between 45 and 60 minutes, either in person or online with the RC, after completing the WSTP intervention. The interview guide will be created by the study team using an iterative process following an appropriate framework (eg, International Classification of Functioning, Disability, and Health) [32]. Questions will explore expectations with wheelchair use, previous experiences with using a wheelchair, and perceptions of wheelchair skills training. In addition, we will explore perceptions of the WSTP (dosage, fatigue, training approaches, and usefulness), experiences with manual wheelchair service provision, satisfaction with manual wheelchair use, and social participation. The interview guide will be pilot tested with 1 person with ARSACS and 1 person with MD1. The interviews will be audio recorded.

### Data Analysis

Summary statistics (means, standard deviations, frequencies, and percentages) will be used to describe the sample. Details of adherence and intervention fidelity will be summarized. Assumptions for parametric testing of quantitative data will be verified, and the data will be screened for outliers [45]. An intention-to-treat analysis will be prioritized (with a per-protocol analysis reported depending on adherence and fidelity), and missing data will be addressed using multiple imputation methods for repeated measures of longitudinal data [46].

To address the primary objective, T2 WST performance scores will be compared between the experimental and control groups using ANCOVA or its nonparametric equivalent, with baseline scores used as the covariate. Variance (*R*^2^), statistical significance (95% CI), and effect sizes (partial η^2^) will be assessed using sums of squares methods. Secondary objectives will be evaluated using linear mixed-effect models, which are flexible to explore within-participant mixed/random effects [47].

Post-hoc exploratory analyses will investigate the influence of age, gender, diagnoses (ataxia or dystrophy), and manual wheelchair experience on effect size using linear mixed-effect models. Comparisons between WST-Q-M within-participant change scores for the experimental group (T1-T2) and WST-Q-M within-participant change scores after training in the control group (T2-T3) will be explored using *t* tests. Sensitivity analyses will be conducted on demographic variables (eg, age, sex, gender, and diagnoses) to generate hypotheses and inform future investigations.

A reflexive thematic analysis will be used to explore perceptions of the influence of manual wheelchair training on mobility, participation, and quality of life according to an appropriate framework (eg, International Classification of Functioning, Disability, and Health) [48]. Interviews will be transcribed verbatim, and researchers will familiarize themselves with the data by rereading and relistening to interviews. Using NVivo software (version 14; Lumivero), transcripts will be coded line-by-line. Then, the codes will be grouped into similar ideas, and themes will be developed using an inductive approach. Member checking will be performed with participants throughout data analysis, with the citizen partner actively involved in data interpretation and synthesis to enhance confirmability. Final themes and subthemes will be verified with participants and the research team. To further enhance trustworthiness according to Ahmed (2024) [49], the credibility of data interpretation will be strengthened through triangulation with quantitative data. Quantitative and qualitative data will then be integrated using joint display diagrams to merge, compare, and relate findings to better understand how subjective perceptions align with measured improvements [50]. We will also explore why some participants may experience better outcomes or how people deal with challenges. Additionally, we will remain aware of potential personal biases regarding our perception of the effectiveness of the intervention. Finally, findings on the facilitators and barriers to training may be transferable to individuals with other neuromuscular disorders who share similar symptoms and functional characteristics [49].

## Results

The study received ethical approval in July 2024 and recruitment started in January 2025. A graduate student, RA, and RC have been recruited and trained. Data collection began in February 2025, and the anticipated completion is February 2026.

## Discussion

### Overview

The need for improved manual wheelchair skills assessment and training in ARSACS and other neuromuscular disorders was observed and documented by our team. Given the strong documented evidence among heterogenous populations of adults [22,23], we hypothesize that an evidence-based standardized manual wheelchair skills training program will improve manual wheelchair skill performance for people with hereditary degenerative disorders such as ARSACS and MD1. This paper describes the protocol for a randomized waitlist controlled trial to evaluate the efficacy of the WSTP on manual wheelchair performance in adults with ARSACS or MD1 and explore the influence of training manual wheelchair skills capacity, manual wheelchair use self-efficacy, manual wheelchair mobility, and satisfaction with participation.

The United Nations declared mobility a basic human right [51]. For people with neuromuscular and hereditary degenerative disorders such as ARSACS and MD1, manual wheelchairs are commonly provided to facilitate self-directed mobility when walking becomes difficult or not possible. Similar to riding a bike, using a manual wheelchair requires skill development and practice over time in indoor and outdoor environments and during real-life situations. ARSACS and MD1 are highly prevalent in the province of Quebec, Canada. This geographic distribution has positioned our team to explore longitudinal aspects of mobility and participation in these 2 specific disorders, which may be generalized in the future to other neuromuscular disorders (eg, spinal muscle atrophy and muscular dystrophy).

Previous studies have documented the clinical significance of learning just one manual wheelchair skill, which could mean the difference between leaving the house independently to get groceries or relying on another person to complete this instrumental activity of daily living [27]. For example, learning how to do a transient tip (ie, popping the small front caster slightly off the ground to get over a door threshold, may make the difference between being able to leave the home or not). Once in the community, learning various approaches to opening heavy doors, maneuvering within small spaces, and navigating irregular surfaces and curbs may make the difference in being able to go to school independently, use public transit, participate in the workforce, or socialize with friends. Given that people with ARSACS have been shown to have very low manual wheelchair skills compared to other adult manual wheelchair users [16], this study has the potential to have major implications on mobility, participation in social and vocational activities, and quality of life for people with ARSACS and MD1.

Establishing the effect size in an RCT will position the research team to work with clinical partners to implement manual wheelchair skills training and evaluation into clinical practice. Given the proximity of the researchers and clinicians involved in this study (ie, the research center and rehabilitation center where outpatient services are provided are in the same building), this trial has the potential to catalyze change in manual wheelchair service provision for people with ARSACS and MD1. Implementing manual wheelchair skills training and evaluation into clinical practice would ensure that training starts early, specifically in the fundamental skills (eg, propulsion and maneuvering) and indoor skills that are required for getting around at home, school, and work. The training of various approaches needed for community and advanced skills (eg, ascending curbs and steep ramps) may be customized based on disease progression, manual wheelchair configuration, and personal experiences. This, in turn, could reduce risks of acute (eg, tips and falls) and overuse (eg, repetitive strain due to poor propulsion techniques) injuries [52-54]. Booster sessions may be provided to people on an as-needed basis, such as after accidents or changes in manual wheelchairs (a service covered by the provincial health care system in Quebec every 5 years). Moreover, optimizing manual wheelchair skills may reduce caregiver burden among those who assist with mobility to accomplish activities of daily living and social participation, roles that are commonly filled by parents, spouses, and other family members. Future studies may explore facilitators and barriers to implementing manual wheelchair skills evaluation into practice with clinicians and managers.

Finally, the collaborations with clinicians, a special interest group in neuromuscular disorders (Muscular Dystrophy Canada), and a citizen partner who has lived experiences with ARSACS and manual wheelchair training research will enhance the credibility of the research findings and increase the likelihood of successful future implementation. New research questions may be developed based on the needs identified by these groups.

A multifaceted knowledge translation strategy will be used to increase awareness and knowledge to change practice for manual wheelchair skills training for people with ARSACS and DM1. Targeted materials for health care professionals will include infographics, podcasts, and video capsules that will be launched through the international PROSPAX (Progression Chart of Spastic Ataxias) initiative on recessive ataxias and neuromuscular disorders (Gagnon), including Rare Disease Day. Materials will be shared through clinical and community platforms including websites and social media of Muscular Dystrophy Canada, Groupe de Recherche Interdisciplinaire sur les Maladies Neuromusculaires, and the Wheelchair Skills Program. Lunchtime presentations and manual wheelchair training workshops will be planned with clinicians. Academic dissemination will include a minimum of 2 peer-reviewed articles and 2 national/international conferences. Led by the corresponding author (KLB), all study investigators will have access to the final data set and will be eligible for authorship.

This study has limitations that should be considered. Although the sample size is powered for the primary outcome, we do not have the power to detect changes in secondary outcomes or for subgroup analysis to explore the influence of potentially confounding factors. Considering the population under study, there may be an increased risk of attrition due to fatigue or difficulty in scheduling training/testing sessions within the desired time frame. We hope to mitigate these issues by offering validated questionnaire versions of tests and by providing training in the community to overcome transportation challenges associated with commuting to a research center. Given that wheelchair configuration and positioning are outside the scope of the WSTP, we will not consider wheelchair configuration or positioning in this study. However, these factors may have an impact on manual wheelchair skill performance and capacity, and ultimately on the efficacy of the intervention.

### Conclusion

The results of this randomized waitlist-controlled trial will confirm whether manual wheelchair skills training can improve self-directed mobility and related outcomes for people with ARSACS and MD1. The findings may help guide clinical practice toward manual wheelchair skills training for understanding potential influences on manual wheelchair mobility among people living with neuromuscular disorders.

## Appendix

Multimedia Appendix 1 Peer-review report by the Muscular Dystrophy Canada.

## Abbreviations

ANCOVA: analysis of covariance
ARSACS: autosomal recessive spastic ataxia of Charlevoix-Saguenay
CIUSSS-CN: Centre Intégré Universitaire de Santé et de Services Sociaux de la Capitale-Nationale
CONSORT: Consolidated Standards of Reporting Trials
MCID: minimally clinically important difference
MD1: myotonic dystrophy type 1
NP: no part
PROSPAX: Progression Chart of Spastic Ataxias
RA: research assistant
RC: research coordinator
RCT: randomized controlled trial
TE: testing error
WheelCon-M: Wheelchair Use Confidence Scale for Manual Wheelchair Users Short Form
WST: Wheelchair Skills Test
WST-Q-M: Wheelchair Skills Test Questionnaire for Manual Wheelchair Users (version 5.4.2)
WSTP: Wheelchair Skills Training Program

## Data Availability

Deidentified data will be made available to the public upon request to the corresponding author (KLB).
