# Supplementary material for: Efficacy of Manual Wheelchair Skills Training for Improving Skills and Confidence in People With Hereditary Degenerative Disorders: Protocol for a Sequential Multimethods Study
Source: JMIR Res Protoc. 2025 Jul 31;14:e66974. doi: 10.2196/66974 (PMC12355141; doi:10.2196/66974)
Supplement: Multimedia Appendix 1 [file resprot_v14i1e66974_app1.pdf]

MUSCULAR DYSTROPHY CANADA
DYSTROPHIE MUSCULAIRE CANADA

Ignited by passion, fueled by hope.

Krista Best Ph.D., M. Sc., B. Sc.
Assistant Professor, Research Scientist
Laval University (Université Laval, Québec) Quebec, Québec, Canada
(Application ID: 1270961 Clinical Research Grant)

Application Title:
Efficacy of wheelchair skills training to improve mobility for people with ARSACS and DM1.

About the Review Process
Applications in this competition were reviewed in two phases:
1. Independent reviews were conducted by a minimum of two medical and scientific reviewers.
2. Applications were then discussed with a full review panel with all reviewers in the competition.

A consensus score was determined and all reviewers voted to determine the ranking relative to all received applications.

Strengths:

- Overall, reviewers found the study to be well-described with specific aims, methods that are well matched to the aims and as described has high likelihood of completion. Reviewers also noted the relevance of the study as it addresses a meaningful issue for daily living for this population. Reviewers found the methods of a randomized control trial, strong randomization, pre-established power and numbers justified, blinded pre-post assessment, 3-month retention assessment to be impressive and strong. The reviewers also liked the use of qualitative data to complement the quantitative data. Reviewers were also impressed with the experience of the team and noted the successes of the study team. There is also a well-detailed knowledge translation plan which reviewers commended.

Weaknesses:

- Reviewers noted the inclusion of only one patient partner to be a weakness as the inclusion of more will allow for better support and flexibility. Reviewers also wondered why an ANCOVA is being used for analysis rather than a regression model and were also concerned about the lack of rigour and analysis approach described in the qualitative methods section.

Recommendations:
Reviewers recommend including a justification for the use of an ANCOVA rather than a regression model for analysis. Reviewers also recommend elaborating on the qualitative methods, specifically on what type of analysis approach will be used (e.g., interpretive, descriptive, etc.). Reviewers also recommend having more than one patient partner as previous literature on patient engagement suggests high levels of burden on patient partners at times and need for flexibility in their work.

Thank you,

Homira Osman, PhD
Vice President, Research and Public Policy

Muscular Dystrophy Canada | 40 Eglinton Ave East, Suite 500 | Toronto, ON M4P 3A2
Tel. 1-800-567-2873 x9037. **Fax** 416-488-0107 *homira.osman@muscle.ca* | **muscle.ca**
